# Pupil Dynamics Reflect Behavioral Choice and Learning in a Go/NoGo Tactile Decision-Making Task in Mice

**DOI:** 10.3389/fnbeh.2016.00200

**Published:** 2016-11-01

**Authors:** Christian R. Lee, David J. Margolis

**Affiliations:** Department of Cell Biology and Neuroscience, Rutgers, The State University of New JerseyPiscataway, NJ, USA

**Keywords:** sensory discrimination, learning, locus coeruleus, norepinephrine, pupillometry, reward

## Abstract

The eye’s pupil undergoes dynamic changes in diameter associated with cognitive effort, motor activity and emotional state, and can be used to index brain state across mammalian species. Recent studies in head-fixed mice have linked arousal-related pupil dynamics with global neural activity as well as the activity of specific neuronal populations. However, it has remained unclear how pupil dynamics in mice report trial-by-trial performance of behavioral tasks, and change on a longer time scale with learning. We measured pupil dynamics longitudinally as mice learned to perform a Go/NoGo tactile decision-making task. Mice learned to discriminate between two textures presented to the whiskers by licking in response to the Go texture (Hit trial) or withholding licking in response to the NoGo texture (Correct Reject trial, CR). Characteristic pupil dynamics were associated with behavioral choices: large-amplitude pupil dilation prior to and during licking accompanied Hit and False Alarm (FA) responses, while smaller amplitude dilation followed by constriction accompanied CR responses. With learning, the choice-dependent pupil dynamics became more pronounced, including larger amplitude dilations in both Hit and FA trials and earlier onset dilations in Hit and CR trials. A more pronounced constriction was also present in CR trials. Furthermore, pupil dynamics predicted behavioral choice increasingly with learning to greater than 80% accuracy. Our results indicate that pupil dynamics reflect behavioral choice and learning in head-fixed mice, and have implications for understanding decision- and learning-related neuronal activity in pupil-linked neural circuits.

## Significance Statement

The head-fixed mouse is an important model system in neuroscience research for relating behavior to the function of neural circuits. Previous studies have shown that video recordings of fast changes in pupil diameter can be used in head-fixed mice to infer brain state underlying behavioral arousal and movement. Pupil measurements are useful because they are non-invasive, can be combined with other types of recordings, and can be repeated longitudinally on the same subject. This study reports pupil dynamics related to learning a Go/NoGo decision-making task in mice. The results show novel information on the choice-specificity of pupil dynamics, and have implications for inferring the function and plasticity of pupil-linked neural circuits.

## Introduction

It has long been appreciated that changes in pupil diameter in humans can reflect cognitive processes such as mental effort, arousal and aspects of decision-making (Kahneman and Beatty, 1966; Richer and Beatty, 1987; Einhauser et al., 2010; de Gee et al., 2014; Murphy et al., 2014b). Accordingly, in addition to its modulation by ambient light levels, pupil diameter has been proposed as a proxy for cognitive- or behavior-related neural activity. Recordings in non-human primates have found a close relationship between fluctuations in pupil diameter and the activity of noradrenergic locus coeruleus (LC) neurons, as well as distributed pupil-linked cortical and subcortical brain areas (Aston-Jones and Cohen, 2005; Joshi et al., 2016). Recent studies in mice have provided extensive evidence that pupil diameter is a useful biobehavioral index of arousal that closely tracks global brain state and the activity of specific types of cortical neurons (Reimer et al., 2014; McGinley et al., 2015a,b; Vinck et al., 2015). However, the relationship between pupil dynamics and more complex learned behaviors in mice still needs to be determined.

There has been considerable interest in investigating the role of LC and pupil-linked arousal systems in different phases of learned behaviors, including cue-reward association and decisions to initiate (Go) and to withhold (NoGo) actions. LC neurons can be phasically activated by salient cues in primates (Aston-Jones et al., 1994; Clayton et al., 2004; Kalwani et al., 2014; Bouret and Richmond, 2015; Varazzani et al., 2015) and in response to orienting cues and rewarded stimuli in rats (Bouret and Sara, 2004). In contrast, LC neurons do not exhibit activation in response to an unrewarded stimulus (Bouret and Sara, 2004) or during decisions to withhold action (Kalwani et al., 2014). Based on these studies showing task-related LC neuron activity, we reasoned that a Go/NoGo decision-making task would be a useful paradigm to investigate the relationship between pupil dynamics and behavioral choice.

Head-fixed mice can be trained to perform various whisker-based behavioral tasks, enabling the study of learning-related neural activity that would be otherwise difficult to interrogate (O’Connor et al., 2009; Huber et al., 2012; Margolis et al., 2014; Chen et al., 2015; Peron et al., 2015; Park et al., 2016). In the Go/NoGo tactile decision-making task of Chen et al. (2013a,b), mice learn to discriminate between two or more textures presented to the whisker by licking (Go) for water reward in response to one of the textures, and withholding licking (NoGo) in response to the distractor textures. We used this task to determine whether pupil dynamics are associated with specific aspects of task performance related to sensory cues, cue-driven behavioral responses (licking), cue-driven behavioral response inhibition (not licking), as well as behaviors such as whisking and licking performed outside of the task structure.

Our results show that task-related pupil dynamics depend on the trial-by-trial behavioral responses, in a stimulus-independent fashion. Furthermore, longitudinal measures show that pupil dynamics become more pronounced across trial types with learning. These results suggest that pupil dynamics reflect choice- and learning-related cognitive processes in mice and have implications for the engagement of arousal systems, including noradrenergic LC neurons, during specific Go and NoGo components of decision-making tasks and at different stages of learned behaviors.

## Materials and Methods

### Animals

All procedures were carried out with the approval of the Rutgers Institutional Animal Care and Use Committee (protocol 13-033). Wild type mice (C57BL/6J; 5 male, 1 female) were purchased from Jackson Laboratory (stock number 00664) and were 63–79 days old at the time of surgery.

### Surgery

Mice were fitted with a custom head post using methods similar to those described previously (Margolis et al., 2012; Chen et al., 2013b). Briefly, mice were anesthetized with isoflurane (4% induction, 0.8–1.5% maintenance) and placed on a feedback controlled heating blanket maintained at 36°C (FHC) mounted on a stereotaxic frame (Stoelting). After the skull was cleaned, light-curable bonding agent (iBond, Heraeus Kulzer) followed by dental cement (Tetric Evoflow, Ivoclar Vivadent) were applied to the surface of the skull. A custom aluminum head post (weight, <1 g) was cemented to the right side skull. After surgery, mice were housed on a reversed light cycle (lights off from 08:00 to 20:00) and had free access to food and water. Following a recovery period of 1 week, mice were handled daily and acclimated to head fixation for an additional week by placing them within a tube (14 cm length, 3.5 cm inner diameter) attached to a custom platform (16.75 cm length, 12.25 cm width) and bolting the head post to a crossbar. Four to 7 days before beginning experiments, daily water intake was limited to 1–2 mL per mouse in order to motivate performance of the behavioral task described below. Body weight was measured once prior to water restriction and daily thereafter. Mice exhibited an average decrease in body weight to 88.2 ± 1.2% of their original weight, consistent with levels of restriction used to motivate behavior (Guo et al., 2014). All handling and behavioral experiments were conducted during the dark phase of the light cycle.

### Pupil and Whisker Imaging

During behavior and other imaging experiments, mice were head-fixed on a holder mounted on an immobile platform and the pupil illuminated with infrared light (740 nm). This illumination did not affect pupil diameter. Whiskers were also illuminated with infrared light (850 nm) during sessions with simultaneous whisker imaging. Behavioral sessions and associated imaging took place in a darkened room, however some ambient illumination (3.48 lux) was present as we found that the pupil became maximally dilated and adynamic in complete darkness. An Allied Vision Technologies Pike F-032 camera was used to image the pupil at 50 frames per second. Whiskers were imaged at 500 frames per second using a Photonfocus DR1 camera. Frames were triggered externally by a Master 9 pulse generator (AMPI). Pupil and whisker data were acquired using Streampix (Norpix) software.

### Texture Discrimination Task

**Figure 1:**
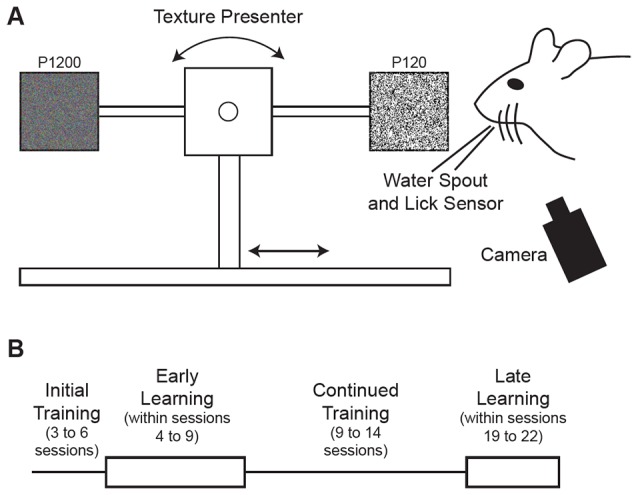
**Behavioral task and training schedule. (A)** Schematic illustration of the tactile decision-making task where mice were trained to discriminate between two textures of different roughness presented to the mouse’s whiskers on a motorized stage. Mice were trained to lick a water spout in response to a Go texture (120 grit sandpaper) and withhold licking in response to the NoGo texture (1200 grit sandpaper). Hit trials, where the mouse licked in response to the Go texture, were rewarded with water delivery through the water spout positioned near the mouse’s mouth. Textures were rotated for each trial after being moved away from the mouse’s whiskers. **(B)** Schematic diagram of training schedule. Mice were initially acclimated to the training setup in 3–6 sessions where the textures were presented with a high probability of the Go texture. Early learning data came from trials within sessions 4–9. Mice were trained in the task for an additional 9–14 sessions. Late learning data came from trials within sessions 19–22.

Head-fixed mice were trained to use their whiskers to discriminate between two textures which were automatically presented in random order using custom software in LabVIEW (National Instruments) operating a linear stage and stepper motor similar to that described previously (Chen et al., 2013a,b; Figure 1A). The water delivery spout was connected to a piezo film sensor that was used to detect licks. Mice were trained to lick a water delivery spout when presented with the Go texture (120 grit sandpaper; P120) and withhold licking when presented with the NoGo texture (1200 grit sandpaper; P1200). Trials began with a 3.05 s baseline, during which the texture was advancing toward the mouse’s whiskers for the final 2.05 s. Correctly licking in response to the Go texture (Hit) within the time the texture was presented (1600–1800 ms after the texture in time) elicited a water reward (5 μl) while incorrectly licking in response to the NoGo texture False Alarm (FA) resulted in delivery of white noise and time out (8000–10000 ms) before the next trial. Withholding licking in response to the NoGo texture Correct Reject (CR) and failing to lick in response to the Go texture (Miss) elicited no water reward and no white noise/time out. Texture presentation was accompanied by a cue tone and reward presentation triggered a reward tone. Sessions were limited to 127 trials, but the session was ended prior to 127 trials if the mouse was no longer performing the task, as indicated by 2–5 consecutive Miss trials.

Behavioral training began with 3–6 initial sessions (Figure 1B) where the Go texture was presented with a high probability (70.2 ± 3.1% of trials; range 62.4–76.4%) to encourage mice to lick. In these initial training sessions, reward was given in some trials (experimenter initiated) to encourage licking, even if the mouse failed to lick in response to the Go texture. Mice were trained twice daily in most cases, however three of six mice completed one session on the initial training day due to limited responding. To determine how learning influenced task-related pupil dynamics over time, we grouped data into early and late learning categories (Figure 1B). Early learning data was acquired from trials occurring in behavioral training sessions 4–9, as indicated in Table 1, to account for performance differences between subjects. In early learning trials, the Go texture probability was reduced to between 50.0–61.9% for four of the six mice, while two mice required a greater Go texture probability to encourage responding (78.3 and 82.6%). Training on the task continued for an additional 9–14 sessions, and late learning data was acquired from sessions 19–22 (indicated in Table 1 for each subject). In late learning sessions the Go texture was presented with an average probability of 46.3 ± 1.6% (range 41.2–52.1%).

**Table 1:** **Dataset for early and late learning by subject**.

Mouse	Early learning sessions	Number of early learning trials	Late learning sessions	Number of late learning trials
A	6	97	21, 22	210
B	6, 7	151	21, 22	243
C	4, 5, 6	351	19, 21	238
D	4, 5, 6, 9	446	19, 21	247
E	4, 5, 6, 9	334	19, 21	237
F	7	98	22	114

### Pupil Analysis and Whisker Tracking

Pupil movies were converted to tiff stacks and whisker movies were converted to AVI format. Pupil movies were read into MATLAB (MathWorks) and the pupil automatically thresholded and segmented using custom-designed routines. Briefly, image intensity values were adjusted equally for all movies in a session using empirically determined parameters that would result in reliable pupil segmentation by the algorithm. The adjusted stacks were then converted to binary stacks that largely segmented the pupil from the rest of the image. The pupil was fully segmented by creating a mask corresponding to a region closest to the center of the image and of size that approximated the pupil diameter. The pupil diameter was measured for each frame by detecting the left and right edges of the mask corresponding to the pupil and calculating the distance between edges in pixels. Pupil measurements during blinks were automatically excluded from analysis and measurements were not adjusted based on changes in eye position. The accuracy of algorithmically derived pupil measurements was determined by comparing a subset of those measurements with pupil measurements that were acquired manually using ImageJ^1^ in 12 representative frames from each mouse (*n* = 6 mice). Measurements obtained using both of these methods showed close correspondence (average pupil diameter 241.8 ± 15.4 pixels with algorithmic measurement and 242.6 ± 15.1 pixels with manual measurement). Pupil diameter is expressed in pixels, or as a percent change in diameter from baseline (defined as the average of the first 50 frames [1 s] of each trial). For group data, mean response profiles were calculated for each trial type for each mouse and then averaged to obtain the overall group mean ± SEM.

Whiskers were tracked and the average whisker angle measured using freely available software implemented in MATLAB (Knutsen et al., 2005).

### Data Analysis

For calculation of cross correlation between average whisker angle and pupil, whisking data from each mouse was temporally downsampled from 500 to 50 frames/s to match the pupil data, and cross correlation calculated using MATLAB’s xcov function.

For onset time and response-operator characteristic (ROC) analysis, pupil diameter traces were interpolated (using MATLAB’s spline function) and smoothed with a 5-frame width, 3-pass boxcar filter (using fastsmooth.m, available at www.mathworks.com/matlabcentral/fileexchange/19998-fast-smoothing-function). Onset of pupil dilation was measured from the average of interpolated and smoothed traces for each subject. Onset time was defined as the first frame greater than 10*SD of the pre-stimulus baseline.

ROC analysis was used to measure the accuracy with which pupil dynamics discriminate behavioral trial type. ROC analysis was performed as in previous work (O’Connor et al., 2010; Chen et al., 2015) on all single-trial data for each subject using interpolated and smoothed traces. One of six mice from late-learning data, and six of three mice from early learning data were excluded from ROC analysis because of low numbers of certain trial types. Discrimination accuracy was based on the similarity of the pupil data in each individual trial to the mean pupil data for each trial type. For each trial, the dot product similarity to the mean of each trial type was calculated. Each trial was classified as one trial type or the other if the difference in dot products exceeded a criterion value. An ROC curve was constructed by varying the criterion value and plotting the probability that a trial of a given trial type exceeded the criterion value against the probability that a trial of the other trial type exceeded the criterion value. Discrimination accuracy was defined as the area under the ROC curve. To generate time-resolved accuracies, the above procedure was performed separately for each 10 frame (0.2 s) time bin. Above chance discrimination was defined by a permutation test. Chance accuracy was calculated by performing ROC analysis 1000 times on data with shuffled trial type labels. Accuracy values above the 95th percentile of shuffled data were defined as discriminating above chance.

### Statistics

Group data are presented as mean ± SEM. Statistics were calculated using either MATLAB or SAS (SAS Institute). Data were compared using paired or unpaired *t*-tests or one way repeated measures analysis of variance followed by paired contrasts as appropriate. Behavior data was compared using McNemar’s test. In all cases tests were performed with significance at *p* < 0.05.

## Results

### Behavioral Performance in The Tactile Decision-Making Task

Mice (*n* = 6) were initially acclimated to the behavioral task for 3–6 sessions. Following this initial training, mice reliably licked in response to the Go texture, and frequently in response to the NoGo texture as well. Early learning data were acquired from 1477 trials within behavioral sessions with stable responding as shown in Table 1. In these trials, the Go texture was presented in 62.7 ± 5.5% of trials while the NoGo texture was presented in the remaining 37.3 ± 5.5% of trials. Mice correctly licked in response to the Go texture (Hit) or withheld licking in response to the NoGo texture CR in 58.9 ± 3.3% of trials. In early learning, most trials resulted in either Hit or FA outcomes (Table 2).

**Table 2:** **Behavior summary by response type in early and late learning**.

Correct		Incorrect		Hit		False alarm		Correct reject		Miss	
Early	Late	Early	Late	Early	Late	Early	Late	Early	Late	Early	Late
58.9 ± 3.3	71.6 ± 4.9	41.1 ± 3.3	28.4 ± 4.9	38.3 ± 5.8	41.2 ± 2.6	16.7 ± 3.7	23.3 ± 3.4	20.6 ± 4.9	30.4 ± 3.4	24.4 ± 3.6	5.0 ± 1.7

*Values represent the percentage of total trials*.

Training on the tactile discrimination task continued, and late learning data was acquired from 1289 trials within the behavioral sessions shown in Table 1. As training progressed, the percentage of trials with presentation of the Go texture was reduced. In late learning, the Go texture was presented in 46.3 ± 1.6% of trials (53.7 ± 1.6% NoGo). In these sessions, Hit or CR outcomes resulted from 71.6 ± 4.9% of trials. The distribution of correct (Hit or CR) and incorrect (FA or Miss) was significantly different in late learning compared to early learning (McNemar’s test; *S* = 8.2761, *p* = 0.0040, df = 1, *n* = 6 mice), indicating that learning occurred.

### Early Learning Pupil Dynamics

**Figure 2:**
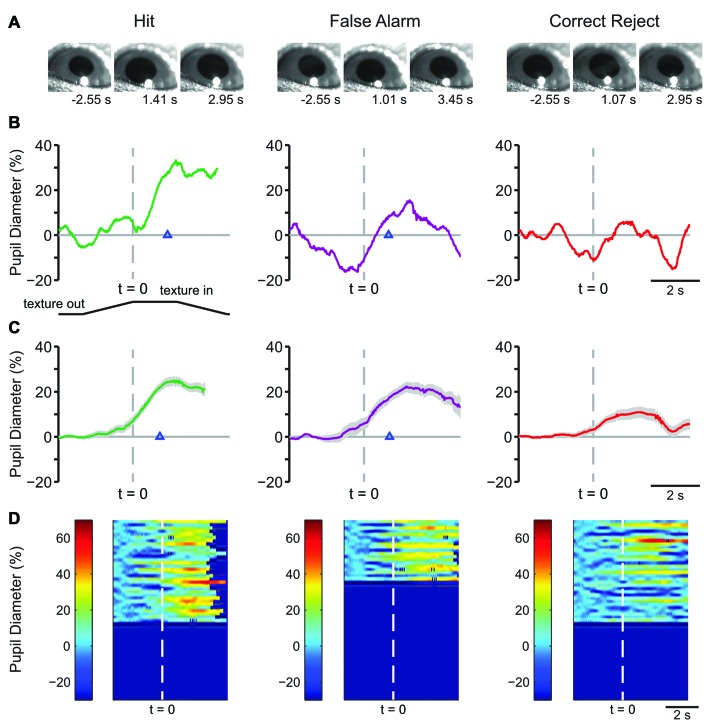
**Early learning pupil dynamics from an example mouse. (A)** Example frames of movies obtained from Hit (left), False Alarm (FA; middle) and correct reject (CR; right) trials in early learning. Frames were obtained at the times indicated from the start of the trial which correspond to a baseline period, the response time and the post response time. **(B)** Pupil diameter vs. time in single trials from an individual mouse. In this and the following figures of a similar type, the position of the texture is schematized below and angled lines indicate when the texture was moving. The time where the texture reached its final position is indicated by the dashed vertical line. Hit and FA trials were characterized by pupil dilation that began prior to the lick response (arrowhead) and continued to evolve through licking and reward presentation. Example plot of pupil diameter during a CR trial in early learning shows slight pupil dilation around the time of texture presentation. **(C)** Average of all Hit, FA, or CR trials from a single behavioral session from an individual mouse in early learning. Shaded area is SEM. Lick responses (arrowheads) occurred at an average latency of 1.097 ± 0.065 s in Hit trials while incorrect licking responses occurred at an average latency of 1.048 ± 0.068 s in FA trials. The average plot of all CR trials from an individual mouse in this behavioral session reveals the modest pupil dilation that occurred around texture presentation. **(D)** Heat map plots of all trials from a single behavioral session show that the patterns of pupil dilation in each response type was similar among most trials within the session.

Example frames from raw pupil movies are shown in Figure 2A. The frames shown are from a time before the texture began its movement (left), at the response time (time of first lick) for Hit and FA and the average response time for CR (middle), and at a time later in the trial, as indicated (right). Single-trial pupil diameter changes reveal characteristic pupil dynamics in each behavioral response type (Figure 2B), which are also evident in the mean of all trials for a representative mouse (Figure 2C). Both Hit (left) and FA (middle) trials, where the mouse made a lick response, exhibited pupil dilation that began prior to the first lick and continued to evolve through the licking. CR trials, where the mouse correctly withheld licking in response to the NoGo texture, were associated with small amplitude pupil dilations around the time of texture presentation followed by constriction back toward baseline. In this session, the response time in Hit trials, measured from the time at which the texture stopped at its final position and, was 1.097 ± 0.065 s, and that for FA trials was 1.048 ± 0.068 s. Miss trials are not shown because there were relatively few (*n* = 10) in this session. Heatmaps of all trials from this behavioral session from a single mouse show that the characteristic pupil changes in each response type are consistent in most trials (Figure 2D).

**Figure 3:**
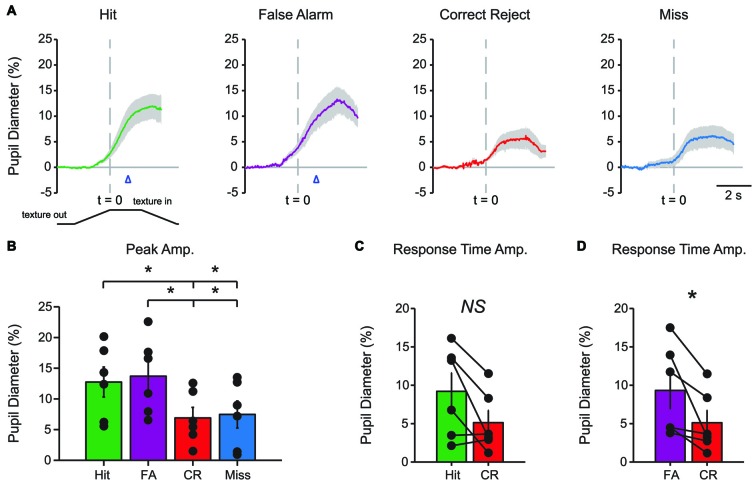
**Mean pupil diameter changes during tactile decision-making in early learning reveal distinct profiles in each response type. (A)** Average plot of all Hit, FA, CR and Miss trials in early learning. In both Hit and FA trials, pupil dilation precedes and continues to evolve through the lick response. The lick response (arrowhead) occurred at an average latency of 1.037 ± 0.057 s in Hit trials and 1.051 ± 0.030 s in FA trials. CR trials were characterized by small-amplitude pupil dilation around the texture presentation time. Miss trials also exhibited small-amplitude pupil dilation that occurred around the texture presentation. **(B)** Peak pupil dilation amplitudes were larger in Hit trials when compared to CR or Miss trials. Peak dilation was also larger in FA trials when compared to CR or Miss trials. **(C)** Pupil dilation amplitudes at the average response time for Hit and CR trials were not significantly different. **(D)** The pupil dilation was significantly different at the average response time in FA trials when compared to CR trials. NS, not significant; *p < 0.05.

Characteristic changes in pupil diameter for each behavioral response type in early learning were apparent in group mean data (Figure 3A). Peak pupil dilations were significantly larger for Hit and FA trials compared to CR and Miss trials (Figure 3B; *F*_(3,15)_ = 7.92, *p* = 0.0021, *n* = 6 mice). Peak dilations in Hit trials were significantly larger than those in CR (*F*_(1,5)_ = 8.73, *p* = 0.0317) or Miss (*F*_(1,5)_ = 7.42, *p* = 0.0416) trials. Similarly, peak pupil dilations in FA trials were significantly larger than those measured in both CR (*F*_(1,5)_ = 14.81, *p* = 0.0120) and Miss (*F*_(1,5)_ = 9.19, *p* = 0.029) trials. These results indicate that behavioral responding (licking) was associated with larger amplitude pupil dilations independent of the tactile stimulus presented, i.e., whether the response was correct (Hit) or incorrect (FA).

We further analyzed whether these differences in pupil dilation were already apparent at the response time (time of first lick). We compared the pupil dilation at the average response time for each mouse in Hit (1.037 ± 0.057 s from texture stop time) and FA (1.051 ± 0.030 s from texture stop time) trials with the pupil dilation in CR trials at the same time point. Miss trials were not included in this analysis. Pupil dilation at the average response time was not significantly different between Hit and CR trials in early learning (Figure 3C; *t* = 2.4791, *p* = 0.0559, df = 5; *n* = 6 mice). In FA trials, pupil dilation at the average response time was significantly greater than that in CR trials (Figure 3D; *t* = 2.8044, *p* = 0.0378, df = 5, *n* = 6 mice).

### Late Learning Pupil Dynamics

**Figure 4:**
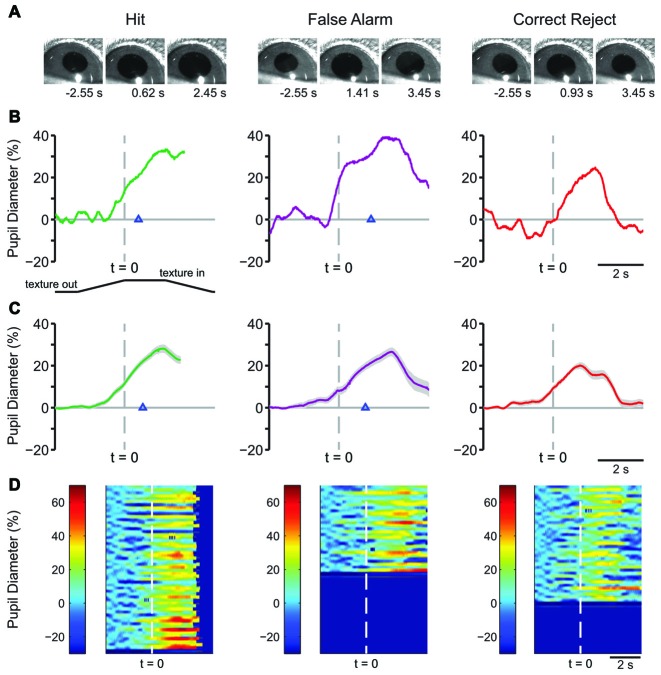
**Characteristic pupil dynamics from a single mouse during tactile decision-making in late learning. (A)** Example frames of movies recorded during Hit (left), FA (middle) and CR (right) trials in late learning. Frames are from times indicated from the start of the trial. **(B)** Plot of pupil diameter during a single Hit, FA, or CR trial. The lick response occurred at the time indicated by the arrowhead in the Hit and FA trials. **(C)** Average of all Hit, FA and CR trials from one mouse in a single behavioral session in late learning. Hit and FA trials were characterized by pupil dilation that both proceeding and continuing through the lick response. Lick responses in Hit trials occurred at an average latency of 0.793 ± 0.022 s and in FA trials at 1.163 ± 0.051 s (arrowhead). The average of all CR trials from this behavioral session reveals transient pupil dilation around the time of texture presentation that quickly returns toward baseline. **(D)** Heat map plots of all trials from the single behavioral session show the patterns in pupil dilation to be a common feature of most trials within each response type.

We continued training the mice on the behavioral task to determine how pupil dynamics changed with further experience. In late learning, behavior-related pupil dynamics became more strongly stereotyped. Example frames from pupil movies are shown in Figure 4A for Hit (left) FA (middle) and CR (right) trials. Plots of single-trial pupil diameter from an example mouse are shown in Figure 4B, and response type averages from a single behavioral session from one mouse are shown in Figure 4C. As in early learning, pupil dilation in Hit and FA trials began near the time of texture presentation, and continued through the lick response and water delivery. CR trials were characterized by pupil dilation around the time of texture presentation followed by a complex “shoulder” waveform, and rapid constriction back to baseline. There were no Miss trials in this behavioral session. Heatmaps of single trials showed consistent patterns of pupil dilation for most trials of each response type (Figure 4D).

**Figure 5:**
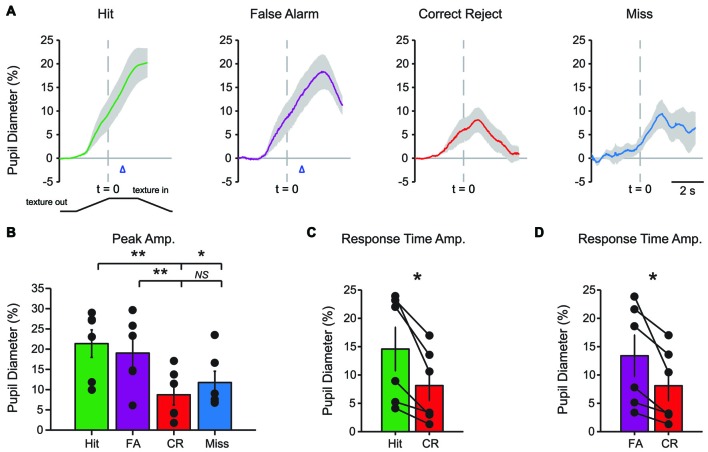
**Mean pupil diameter changes during tactile decision-making in late learning reveal characteristic profiles in each response type. (A)** Hit trials exhibited pupil dilation preceding and continuing through the lick response (arrowhead) which occurred at an average latency of 0.898 ± 0.047 s from the texture stop time. FA trials similarly exhibited pupil dilation both preceding and continuing through the incorrect lick response. Incorrect licking in FA trials occurred at an average latency of 0.920 ± 0.055 s (arrowhead). CR trials were characterized by small-amplitude pupil dilation around texture presentation that rapidly returned toward baseline. Miss trials exhibited small-amplitude pupil dilations following texture presentation that tended to remain through the recording. **(B)** Peak pupil dilation was greater in Hit trials when compared to CR or Miss trials. Peak dilation was also significantly greater in FA trials when compared to CR trials, but was not significantly different than Miss trials. **(C)** At the average lick response time, pupil dilation was significantly greater in Hit trials when compared to CR trials. **(D)** Similarly, pupil dilation was significantly greater at the average lick response time in FA trials when compared to CR trials. NS, not significant; *p < 0.05; **p < 0.01.

Characteristic changes in pupil diameter for each behavioral response type in late learning were apparent in group mean data (Figure 5A), including overall larger amplitude and earlier onset dilations, as well as the more complex waveform for CR responses. Peak dilation amplitudes were significantly different across response types (Figure 5B; *F*_(3,15)_ = 12.39, *p* = 0.0002 *n* = 6 mice). Specifically, when comparing trials with a lick response against those without a lick response, Hit trials had larger peak dilations than CR (*F*_(1,5)_ = 42.75, *p* = 0.0013) and Miss (*F*_(1,5)_ = 9.03, *p* = 0.0299) trials. Peak dilations in FA trials were larger than those in CR trials (*F*_(1,5)_ = 26.82, *p* = 0.0035), but were not significantly greater than those in Miss trials (*F*_(1,5)_ = 4.56, *p* = 0.0858), possibly because of the low number of Miss trials in late learning.

We again compared the magnitude of pupil dilation at the average response time in Hit and FA trials to the pupil diameter measured at the corresponding time in CR trials. In late learning, the magnitude of pupil dilation at the average response time was significantly greater in Hit trials (average response time, 0.898 ± 0.047 s) when compared to CR trials (Figure 5C; *t* = 3.8109, *p* = 0.0125, df = 5, *n* = 6 mice). Similarly, pupil dilation was significantly greater in FA trials (average response time, 0.920 ± 0.055 s) when compared to CR trials at the average response time (Figure 5D; *t* = 3.0848, *p* = 0.0273, df = 5, *n* = 6 mice). Thus, in contrast to results from early learning data, late learning pupil dilations achieved clearer and significant differences by the time of the first lick response.

Overall, these results indicate that pupil dynamics display characteristic patterns of dilation that vary by behavioral response during performance of the Go/NoGo tactile decision-making task. Notably, larger pupil dilations occur during responses that involve licking, independent of the identity of the texture presented, suggesting that pupil dynamics are more closely linked with behavioral response (licking) than sensory cues.

### Learning-Related Changes in Pupil Dynamics

**Figure 6:**
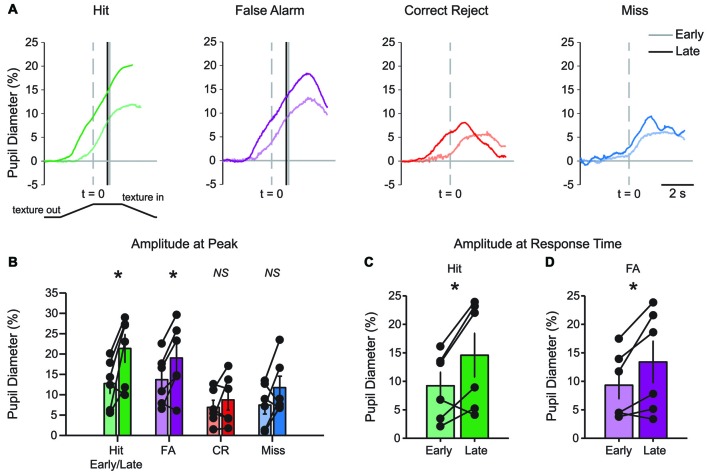
**Learning-related changes in pupil diameter responses during tactile decision-making. (A)** Overlay of average pupil diameter responses in Hit, FA, CR and Miss trials. The average response time is indicated by the solid vertical line for early and late learning in Hit and FA trials. **(B)** Peak pupil dilations were significantly larger in late learning in Hit and FA trials, but were not significantly larger in CR and Miss trials. **(C)** When compared at the average response time in early and late learning, pupil dilation was larger in late learning for Hit and **(D)** FA trials. NS, not significant. *p < 0.05.

The basic patterns of pupil dilation that occurred during each behavioral response type could be resolved in early learning, but the effects became more pronounced in late learning. Comparisons of early and late mean data suggested that pupil dilations in late learning had earlier onset and larger amplitudes (Figure 6A). The time of pupil dilation onset (defined as the first frame greater than 10*SD of pre-stimulus baseline in traces interpolated and smoothed to reduce noise) was significantly advanced in Hit trials (−0.302 ± 0.301 s early and −1.062 ± 0.137 s late; *t* = 2.8522, df = 4, *p* = 0.0463; *n* = 5 mice) and CR trials (0.166 ± 0.223 s early and −0.934 ± 0.351 s late; *t* = 3.9724, df = 4, *p* = 0.0165; *n* = 5 mice). The difference in pupil dilation onset times did not reach significance in either FA trials (−0.254 ± 0.345 s early and −0.61 ± 0.257 s late; *t* = 1.2684, df = 4, *p* = 0.2735; *n* = 5 mice) or Miss trials (−0.366 ± 0.396 s early and 0.15 ± 0.323 s late; *t* = −0.9458, df = 4, *p* = 0.3978; *n* = 5 mice). Peak dilation amplitudes were greater in late learning for both Hit (Figure 6B; *t* = −3.2381, df = 5, *p* = 0.0230; *n* = 6 mice) and FA (*t* = −3.3396, df = 5, *p* = 0.0206; *n* = 6 mice) trials when compared to early learning. Peak dilation amplitudes were not significantly different for early and late learning in either CR trials (*t* = −0.9490, df = 5, *p* = 0.3862; *n* = 6 mice) or Miss trials (*t* = −1.6367, df = 5, *p* = 0.1626; *n* = 6 mice). Pupil dilation at the average lick response time was similarly larger in late learning for both Hit (Figure 6C; *t* = −2.8694, df = 5, *p* = 0.0350; *n* = 6 mice) and FA (Figure 6D; *t* = −2.5945, df = 5, *p* = 0.0486; *n* = 6 mice) trials when compared to early learning. These longitudinal results indicate that learning of the tactile decision-making task leads to larger amplitude task-related pupil dilations that begin earlier within the behavioral trial. While Hit responses grew in amplitude and were earlier onset, FA responses grew in amplitude without a change in onset. CR responses, on the other hand, became earlier in onset, but did not change in amplitude. Miss trials changed in neither onset nor amplitude.

### Pupil Discrimination of Behavior

**Figure 7:**
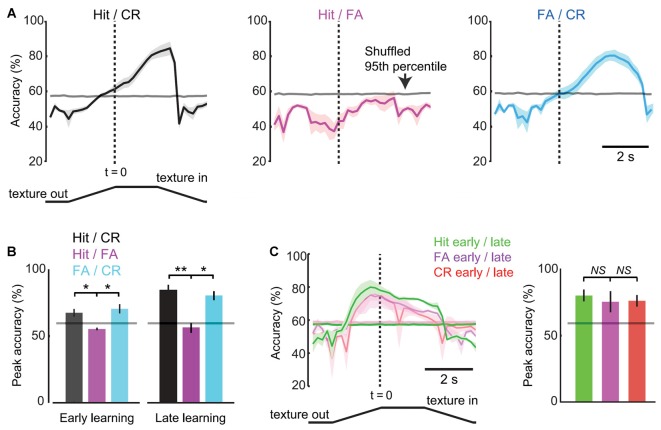
**Response-operator characteristic (ROC) analysis of pupil dynamics and behavior. (A)** Time-resolved discrimination accuracies of pupil dynamics for Hit vs. CR (left), Hit vs. FA (middle), FA vs. CR (right) trials (*n* = 5 mice in late learning sessions). The gray line near 60% represents chance levels defined as the 95th percentile of data shuffled 1000 times. **(B)** Peak accuracy values for trial type comparisons in early and late learning (mean ± SEM; *n* = 3 and *n* = 5 mice, respectively). Horizontal lines depict chance levels. **(C)** Time-resolved discrimination accuracies of pupil dynamics for early vs. late trials (left), with summary of peak accuracies by trial type (right). NS, not significant. *p < 0.05; **p < 0.01.

We performed ROC analysis to determine the accuracy with which single-trial pupil dynamics were able to discriminate behavioral responses. Analysis was performed on each 10-frame (0.2 s) bin to resolve time-varying changes in discrimination accuracy during task performance. In late learning, discrimination accuracy for comparison of Hit and CR trials reached greater than chance values (defined as the 95th percentile of shuffled data) on average 0.6 s before the texture in time (1.4 s after the texture began to translate toward the mouse; Figure 7A, left) and was significantly different from the Hit/FA comparison at this time point (57.9 ± 1.1% vs. 42.1 ± 5.5%, mean ± SEM; *p* = 0.0450, paired *t*-test; *n* = 5 mice). At the response time (time of first lick), discrimination accuracy reached 71.1 ± 3.3% (significantly different from Hit/FA trials; 53.0 ± 2.1%; *p* = 0.0135, paired *t*-test; *n* = 5 mice) before further increasing to its maximal value of 82.8 ± 3.8%. By contrast, pupil dynamics did not discriminate Hit from FA trials at any time points (peak 56.3 ± 3.9% accuracy, mean ± SEM; mean 95th percentile of shuffled data 57.9% at same time point; Figure 7A, middle). Discrimination accuracies for FA vs. CR trials were similar to those for Hit vs. CR trials (Figure 7A, right), and both were significantly greater than Hit/FA accuracies (Figure 7B; *t* = 5.4726, df = 4, *p* = 0.0054, Hit/CR vs. Hit/FA, *n* = 5 mice; *t* = 3.6496, df = 4, *p* = 0.0218, CR/FA vs. Hit/FA, *n* = 5 mice; paired *t*-tests), consistent with the large dilations occurring during both Hit and FA trials. These results indicate that, in late learning, single-trial pupil dynamics predict the behavioral choice (Hit or FA vs. CR) of mice with accuracy well above chance, and do so early within the behavioral trial.

We performed similar analysis of early learning data from 3/5 of the same mice analyzed above (data from two mice were excluded due to insufficient data for single-trial analysis). Peak accuracy values comparing Hit and CR were greater than chance in early learning but significantly lower than accuracy values in late learning (66.7 ± 2.0%, *n* = 3 mice; 82.8 ± 3.8%; *p* = 0.0223, unpaired *t*-test; *n* = 5 mice; Figure 7B), indicating that the accuracy with which pupil dynamics predict behavioral choice increases with learning. To further analyze learning-related changes in discrimination accuracy, we performed ROC analysis on early vs. late learning pupil dynamics of the same trial type. The time-resolved accuracy plots of early/late learning comparisons showed an increase in above chance accuracies that were early and sustained, and similar across response types (Figure 7C; *p* = 0.3807 Hit early/late vs. FA early/late, *n* = 3 mice; *p* = 0.8929, CR early/late vs. FA early/late, *n* = 3 mice; paired *t*-tests). The early increase was present across trial types, consistent with the shorter onset times measured from average pupil dynamics in late learning (above). The sustained high accuracy throughout the time of the trial is likely due to the increased amplitude dilations in late learning. Together, these single-trial analysis results are consistent with the average pupil dynamics of Figure 6, and demonstrate that single-trial pupil data can be used to predict behavioral responses with high accuracy.

### Effects of Reward and Whisking on Pupil Diameter

Recent studies have established a relationship between pupil dilation and periods of movement in mice, including locomotion and whisking (Reimer et al., 2014; McGinley et al., 2015a; Vinck et al., 2015; Mineault et al., 2016). Because different types of movements are involved in performance of the tactile decision-making task, we sought to determine the relative contributions of whisker movement and licking for water reward to the observed task-related increases in pupil diameter. Therefore, in separate experiments, we measured pupil dynamics during task-independent (spontaneous) whisking, task-related whisking and presentation of unexpected water reward.

**Figure 8:**
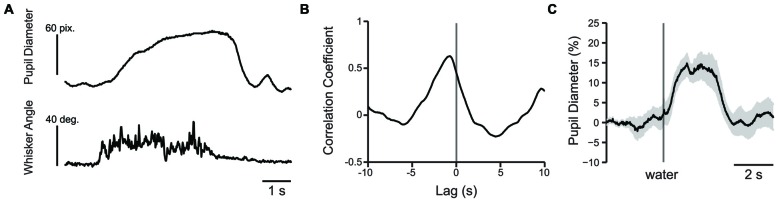
**Pupil dilation occurs in response to whisking or presentation of water reward. (A)** Plot of pupil diameter (50 frames/s) and simultaneously recorded whisker movement (500 frames/s). Note that pupil dilation occurs during whisking. **(B)** An example cross correlation calculated from 60 s of spontaneous pupil diameter and whisker angle data shown in **(A)**. The strongest correlation, in this case −0.63 occurred at −740 ms. **(C)** Water reward induces pupil dilation. Plot of pupil diameter during presentation of an uncued water reward presented at the time indicated by the vertical line shows that presentation of water reward induces pupil dilation. Data is from an average of nine trials and shaded area is SEM.

The change in whisker angle (recorded at 500 frames/s) was cross correlated with simultaneously recorded pupil diameter (recorded at 50 frames/s; *n* = 3 mice; Figure 8A). The maximal cross correlation was 0.56 ± 0.06 at a lag of −693 ± 29 ms (Figure 8B), indicating that pupil dilation follows active whisking, consistent with recent work (Reimer et al., 2014). Given the association of whisking with pupil dilation, we sought to determine whether whisking, or a distinct process such arousal or cognitive load, drove pupil dilation during behavioral performance. We measured the cross correlation between pupil diameter and whisker angle during texture presentation in the 1 s task period immediately preceding the texture stop time in a subset of mice (using data concatenated across trials). Cross correlation values from early and late learning were similar in both strength (*t* = 0.0788; df = 2; *p* = 0.9443; *n* = 3 mice) and lag (*t* = 2.1581; df = 2; *p* = 0.1636; *n* = 3 mice) and were combined. Under these conditions, the maximal cross correlation was 0.185 ± 0.025 at a lag of −340 ± 20 ms, significantly lower than the correlation between pupil diameter and spontaneous whisking in strength (*t* = 6.0484; df = 4; *p* = 0.0038; *n* = 3 mice) and shorter in lag (*t* = −10.0440, df = 4; *p* = 0.0005; *n* = 3 mice). These data suggest that whisking likely contributes to, but does not fully account for, the pupil dilation present during performance of the tactile decision-making task. The task related pupil dilations we observed likely reflect task-related behavioral and/or cognitive processes involved in decision-making, in addition to whisker movements. In separate experiments, we found that presentation of an unexpected water reward was also associated with pupil dilation (Figure 8C). Licking-related pupil dilations likely contribute to the sustained dilations that occur at the end of Hit and FA trials. Taken together, these results suggest that whisking, cognitive factors, and licking all likely contribute to the pupil dilations that occur during performance of sensory-guided decision-making behaviors.

## Discussion

We found that characteristic task-related pupil dynamics are associated with behavioral choice during a tactile decision-making task in mice. As mice learned the task, pupil dynamics became larger in amplitude, earlier in onset and, notably, more highly predictive of the behavioral choice. Our results suggest that pupil dynamics in mice reflect cognitive aspects of task performance, in addition to motor-related changes during whisking and licking, and may be analogous to the task-related pupil changes present in humans and non-human primates.

### The Relationship Between Pupil Diameter and Decision-Making in Mice

A main finding of our study is that distinct pupil dynamics are more closely associated with the type of behavioral response than the type of tactile stimulus presented. The largest amplitude pupil dilations occurred during trials in which licking occurred (both Hit and FA trials), consistent with the occurrence of pupil dilation during various types of movements including locomotion (Reimer et al., 2014; McGinley et al., 2015a; Vinck et al., 2015), whisking and task-independent licking for water (as shown here). We also observed pupil dilations that occurred earlier within trials near the time of tactile stimulus presentation, well before the time of the first lick. These early dilations occurred in all behavioral responses to varying degree, even in Miss trials when mice failed to respond to the Go texture, supporting the idea that pupil-linked arousal systems are activated by sensory cues. However, we also observed pupil dilations that occurred even earlier, coincident with movement of the motorized stage that signaled trial start, and were particularly prominent in late learning conditions, with onset times up to 1 s before stimulus presentation. This result suggests that pupil dilation does not only reflect movement, but could also reflect aspects of anticipation/expectation, motor preparation, or the intention to respond. These task-related early dilations were smaller in amplitude than movement-related dilations and could be related to the small fluctuations between movement bouts observed by Reimer et al. (2014).

Our results suggest that pupillometry may be particularly well suited to investigate certain types of behavioral tasks in mice. Use of the Go/NoGo task allowed us to observe pupil dynamics during sensory-guided response initiation (Go) as well as response inhibition (NoGo). Response inhibition is a more difficult type of decision process to study because it is covert, i.e., not associated with a behavioral response. Pupillometry can be useful in situations such as this, as shown by our results suggesting that it was possible to distinguish CR from Miss trials in late learning by the earlier onset and more complex dynamics present in CR trials. The rapid constriction to baseline in late learning CR trials was relatively time-locked, suggesting that pupil constriction could indicate the timing of the decision to inhibit responding. More broadly, pupil measures could be useful in a decision-theoretic context for inferring other behavioral/cognitive states that are not associated with a specific motor response, such as attention, anticipation, surprise, or decision confidence, and could help to shed light on the relationship between pupil-linked neuromodulator systems and behavioral optimization during learning (Gold and Shadlen, 2007; Dayan and Daw, 2008; Sara, 2009). Furthermore, a recent investigation in non-human primates found that multiple, distributed brain regions, including anterior cingulate cortex (ACC), are active with distinct timing relative to pupil dilation and LC activity (Joshi et al., 2016). The extent of pupil-linked neural circuits in mice remains to be identified, but one intriguing possibility is that pupil dynamics could be related to neural activity in brain regions such as ACC or orbitofrontal cortex that are involved in executive control and other aspects of choice behavior (Kepecs et al., 2008). Such cognitively driven pupil diameter changes in mice await further investigation.

### Pupil Diameter as a Readout of Neuromodulator Systems and Brain State

There is great interest in understanding how changes in pupil diameter relate to behavioral state and brain state, because pupillometry can be performed non-invasively and easily combined with various experimental paradigms. Prominent theories are based on evidence suggesting that pupil diameter tracks changes in the activity of noradrenergic LC neurons in humans and non-human primates (Aston-Jones and Cohen, 2005; Gilzenrat et al., 2010; Murphy et al., 2014a; Varazzani et al., 2015). Behavioral arousal is closely related to LC activity (Samuels and Szabadi, 2008a; Carter et al., 2010; Sara and Bouret, 2012) and pupil dilation in mice (Reimer et al., 2014; McGinley et al., 2015b; Vinck et al., 2015), as well as other neuromodulatory systems including acetylcholine (ACh; Eggermann et al., 2014; Lin et al., 2015; Harrison et al., 2016). Therefore, the task-related pupil dilations that we report are likely correlated with activation of pupil-linked neuromodulator systems, including LC neuron activity, but this remains to be tested by directly recording LC neurons in mice. Our results showing pupil dilations early within the task before stimulus presentation, as well as later in the task during licking and water reward consumption, are consistent with the engagement of LC and other neuromodulator systems during various phases of behavior, including trial onset cues (anticipation; Bouret and Sara, 2004), processing of sensory cues (Aston-Jones et al., 1994) and preparation for upcoming actions (Sara and Bouret, 2012; Varazzani et al., 2015).

Although pupil diameter covaries with LC activity, the mechanism underlying this relationship remains unknown. Pupil diameter and LC activity could be correlated by common afferent input to both LC and nuclei that control pupil diameter (Gilzenrat et al., 2010; McGinley et al., 2015b). There is also some evidence that LC noradrenergic signaling could control pupil diameter more directly. For example, pharmacological increase of LC neuron activity causes pupil dilation in both rats and mice that is abolished by lesion of LC neurons but unaffected by disruption of sympathetic innervation of the eye (Prow et al., 1996; Yu et al., 2004). LC can modulate activity of both sympathetic and parasympathetic input to the dilator pupillae through its innervation of the superior cervical ganglion and Edinger-Westphal nucleus, causing dilation and constriction, respectively (Samuels and Szabadi, 2008b). While the causal relationship between LC and pupil dilation remains undetermined, our results can be interpreted as providing an index of the activation of pupil-linked neuromodulator systems during tactile decision-making and learning in mice. However, determining the precise relationship between pupil dynamics and LC neuron activity during tactile decision making will require direct measurement of LC neuron discharge. This is especially true given that LC neurons exhibit a broad repertoire of signaling capacity that can be encoded through changes in their firing rates and patterns of firing (Aston-Jones and Bloom, 1981; Sara, 2009).

### Implications of Learning-Related Changes in Pupil Dynamics

We found that task-related pupil dynamics changed in both amplitude and timing as mice progressed from early- to late-learning. Peak dilation amplitudes were approximately 50–80% larger on average in late learning for Hit and FA trials compared to early learning, and only slightly increased for CR and Miss trials. Furthermore, pupil dilation began at earlier time points in the trials, up to 0.7–1.0 s earlier on average for Hit and CR trials. Notably, the correct behavioral responses (Hit, CR) rather than the incorrect responses (FA, Miss) were the trials that showed significant learning-related advancement of pupil dilation onset, raising the possibility that earlier engagement of pupil-linked neural systems leads to improved task performance.

These results suggest that learning involves an earlier and stronger recruitment of pupil-linked neural activity during task performance. Recent work indicates that pupil dilation in mice is correlated with a desynchronized cortical state and inversely correlated with the occurrence of hippocampal fast ripples (Reimer et al., 2014; McGinley et al., 2015a,b). Desynchronized cortical states are associated with improved sensory fidelity and behavioral detection (Devilbiss and Waterhouse, 2011; Reimer et al., 2014; Martins and Froemke, 2015; McGinley et al., 2015a; Vinck et al., 2015; Fazlali et al., 2016; Mineault et al., 2016). In our experiments, pupil-linked cortical desynchronization could function to improve texture discrimination by enhancing sensory coding or refining sensorimotor integration processes involved in sensory-guided decision-making. It is likely that increased attention or expectation, which have been associated with heightened arousal and desynchronized cortical states, are important in task learning and may be causally related to the earlier onset pupil dynamics in late learning.

Pupil-linked desynchronized cortical states are also closely associated with increased activation of neuromodulator systems, including norepinephrine from LC (Polack et al., 2013; Fazlali et al., 2016) and ACh from basal forebrain (Goard and Dan, 2009; Eggermann et al., 2014; Lin et al., 2015). Furthermore, the recruitment of LC neurons by salient cues can be modified with training, suggesting that the role of noradrenergic signaling in task performance increases and becomes more important with experience (Martins and Froemke, 2015). This is consistent with our results showing larger pupil dilation in trials with licking responses in late learning (Hit, FA). An interesting possibility is that manipulation of pupil-linked neuromodulator systems could be used to improve learning and behavioral performance. Learning of stimuli presented with induced pupil dilation in humans has been associated with improved learning (Nassar et al., 2012; Hoffing and Seitz, 2015). The causal relationship between pupil-linked neuromodulator systems (including LC) with arousal, learning, sensory discrimination and decision-making could be tested with greater temporal and cell-type specificity in rodent models (Janitzky et al., 2015).

## Conclusion

We have shown that distinct pupil dynamics reflect choice-specific behavioral responses in head-fixed mice performing a tactile decision-making task. Pupil dynamics begin before licking responses and are highly predictive of upcoming behavioral responses to lick (correctly or incorrectly) or to withhold licking (correctly). Task-related pupil dilations became larger and started earlier with learning, suggesting plasticity in the neural mechanisms underlying behavioral choice-related pupil dilation. Given the increasingly appreciated relationships between pupil diameter and cortical and subcortical activity, our results have implications for understanding how pupil-linked neuromodulator systems, including noradrenergic LC neurons, are engaged at specific times during decision-making tasks and learning.

## Author Contributions

CRL and DJM designed research, CRL performed research, CRL and DJM analyzed data, CRL and DJM wrote the article.

## Conflict of Interest Statement

The authors declare that the research was conducted in the absence of any commercial or financial relationships that could be construed as a potential conflict of interest.

## Footnotes

^1^http://imagej.nih.gov/ij/
